# Primary epididymis malignant triton tumor: case report and review of the literature

**DOI:** 10.1186/s40001-015-0172-y

**Published:** 2015-09-21

**Authors:** Lijian Gao, Hualin Song, Kun Mu, Jiaxin Wang, Baoyin Guo, Benkang Shi, Gang Li

**Affiliations:** Department of Urology, Qilu Hospital of Shandong University, Jinan, Shandong Province China; Department of Urology, Dezhou People Hospital, Dezhou City, Shandong Province China; Department of Urology, Second Hospital of Tianjin Medical University, Tianjin Institute of Urology, Pingjiang Road, No 23, He Xi District, Tianjin, 300211 China; The Second Department of Breast Cancer, Tianjin Medical University Cancer Institute and Hospital, National Clinical Research Center for Cancer, Key Laboratory of Cancer Prevention and Therapy, Tianjin, 300060 China; Department of Head and Neck Cancer, Tianjin Medical University Cancer Institute and Hospital, National Clinical Research Center for Cancer, Key Laboratory of Cancer Prevention and Therapy, Tianjin, 300060 China; Department of Urology, Tianjin Baodi Hospital of Tianjin Medical University, Tianjin, 300321 China

**Keywords:** Malignant triton tumor, Epididymis, Pathology, Treatment

## Abstract

**Background:**

Malignant triton tumor (MTT) is a rare and histological complexity characterized by a mixture of peripheral nerve sheath tumors and with rhabdomyoblastic differentiation. It follows a particularly malignant course.

**Case presentation:**

In the present study, we report the first MTT of epididymis. The patient is a 22-year-old male presented with swelling in the left scrotum over a 2-month period. He did not have the history or symptoms of neurofibromatosis type 1. A mass measured about 3 cm × 4 cm was found in the left epididymis by ultrasound and CT scan. It was diagnosed as epididymis tumor and underwent exploration; intraoperative frozen section was diagnosed malignant tumor and treated with radical orchidoepididymectomy. The pathological report was malignant triton tumor. Despite taken high-dose radiation therapy and followed by chemotherapy for four cycles, he was died of progressive disease with multiple metastases 26 months after surgery. The clinic pathologic characteristics and optimal treatment strategy are reviewed.

## Background

Malignant triton tumor (MTT) is a very rare and histological variant of the malignant peripheral nerve sheath tumor with rhabdomyoblastic differentiation. Its incidence is one per 100,000 and usually arising in trunk regions, head, neck [1] and rarely occurs in male genital system especially in epididymis. MTT tends to occur in individuals younger than 35, approximately half to two-thirds of cases occur in the context of neurofibromatosis type 1 (NF-1) [2]. Treatment modalities involved radical surgical resection,adjuvant chemotherapy and radiation. Total resection of the tumor is the most important therapeutically methods. To identify and analyze the clinic pathologic characteristics, therapeutic and the evolution of the disease are important for clinical decision-making.

## Case presentation

**Fig. 1:**
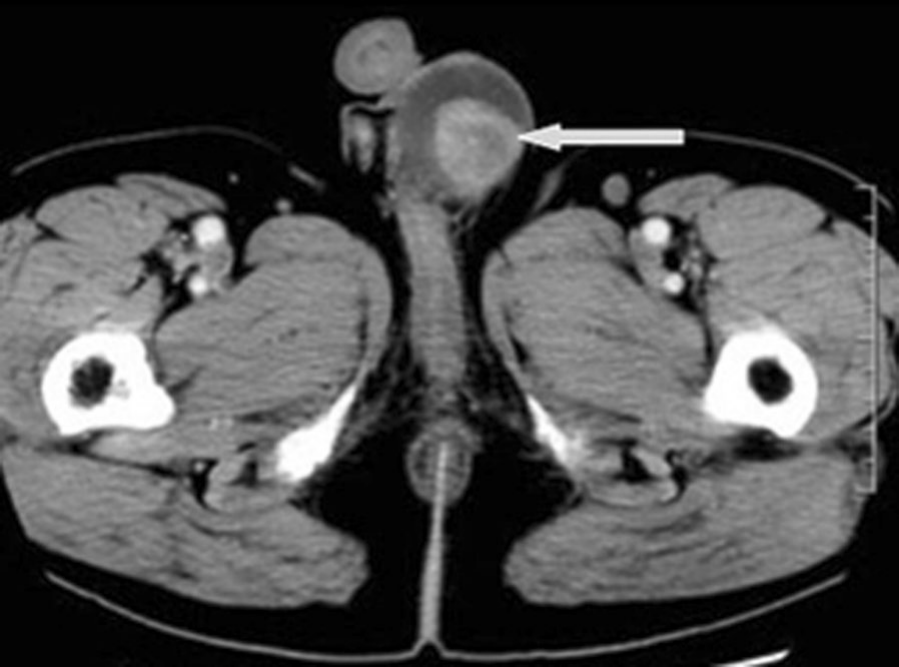
Axial CT image of scrotum revealed a solid mass measured about 3 cm × 3 cm in the left epididymis with regional extension to the testis (*White arrow*)

**Fig. 2:**
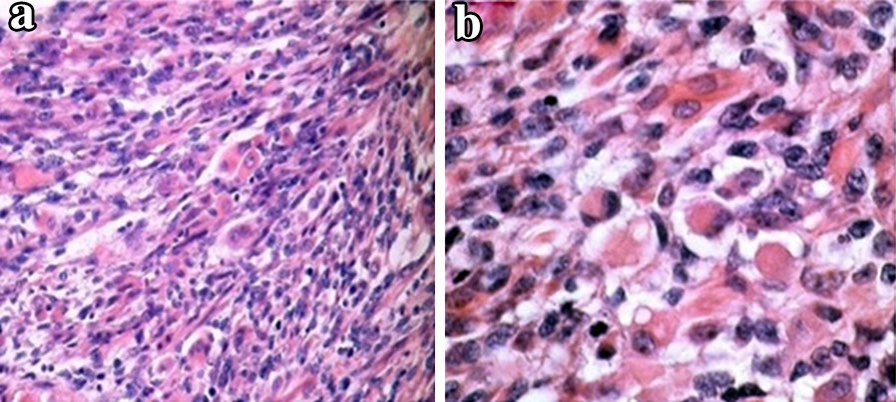
**a** Histologically, malignant peripheral nerve sheath tumors with highly cellular areas neighboring less cellular ones, with dense eosinophilic cytoplasm and cytoplasmic cross striations were scattered through the stroma, which was made up of pleomorphic spindle cells (HE ×100). **b** Nests of cells with rhabdomyoblastic differentiation are seen to lie in a mucoid stroma (HE ×400)

**Fig. 3:**
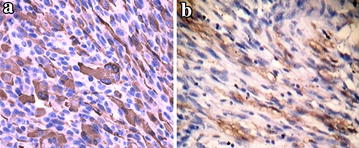
**a** The tumor was immunoreactive for myoid marker Desmin. **b** Immunoreactivity for the neuronal marker S100. **a**, **b** Immunohistochemistry, ×200)

The patient is a 22-year-old male presented with a painless scrotal lump in the left scrotum over a 2-month period. When the patient was admitted to hospital, physical examination revealed a large and palpable scrotum mass in the left side of the epididymis without enlarged lymph nodes. Laboratory studies such as blood counts, lactate dehydrogenase, beta-HCG, and alpha-fetoprotein were normal and did not reveal abnormalities. Ultrasound of the scrotum were performed and revealed a hypoechoic mass measured about 3 cm × 4 cm in diameter with anechoic areas. On computer tomography, a solid mass was found in the left epididymis with regional extension to the testis. There were low density areas surrounding the mass (Fig. 1). A conventional radiographic bone and chest survey also yielded no abnormal findings. Computed tomographic scan of abdomen revealed no evidence of metastasis in local and distant organs. It was diagnosed as epididymal neoplasm and hydrocele. The patient underwent surgical exploration. On operation, bloody hydrocele was seen and a solid rough mass was invaded into the testis. Intraoperative frozen section was diagnosed as a malignant tumor and the patient underwent a radical inguinal orchidectomy. The postoperative course was smooth. Specimen was stained with hematoxylin and eosin. Microscopically, the tumor tissue disclosed a malignant schwannoma with rhabdomyosarcomatous features. Rhabdomyoblasts with dense eosinophilic cytoplasm and cytoplasmic cross striations were scattered through the stroma, which was made up of pleomorphic spindle cells (Fig. 2a, b). The immunohistochemistry was positive for S-100 protein, NF, CD68, Ki67, MYOD1 and Desmin, leading to the diagnosis of MTT (Fig. 3a, b). Histological and immunohistochemical findings supported the diagnosis of malignant triton tumor.

He was treated with four cycles of multi-agent systemic chemotherapy with ifosfamide, vincristine, carboplatin, epirubicin regimen and received 3600 cGy of radiotherapy to the primary site and groin area. The patient had evidence of multi-organ metastasis on clinical or imaging examination during 14th months’ follow-up. Although three-stage and multiple agents chemotherapy were performed, unfortunately, partial response was achieved and the patient died of 12 months later.

## Discussion

Most masses within the scrotal sac are within the testis and neoplastic. Neoplasms arising from epididymis are benign behavioral patterns while on rare occasions are malignant. Tumors occurring in the epididymis region may be clinically indistinguishable from testicular tumors, thus resulting in initial misdiagnosis. Most tumors of this region present as a scrotal mass or swelling, which may be asymptomatic. The preoperative distinction between the benign and malignant epididymis tumor is difficult, as there are no specific tumor markers.

Epididymis tumors are commonly soft-tissue or mesothelial neoplasm in origin. Cystadenomas, papillary tumors and adenomatoid tumor are the most common tumors. It is imperative to consider malignant tumor in the differential diagnosis of all solid tumors of the scrotum. Metastases, particularly from testicular can also occur. MTT is classified as a malignant peripheral nerve sheath tumor with rhabdomyosarcomatous differentiation [3]. The unusual name “triton” was first used in reference to observation of supernumerary limbs containing bone and muscle growing the backs of tritons after the implantation of the sciatic nerve into the soft tissues of the back. The histogenesis of MTT has been extensively debated, it arises along a peripheral nerve, or in a ganglioneuroma, or in a patient with neurofibromatosis type 1 and rhabdomyoblasts arise within the body of the tumor [4]. Hypotheses have been proposed to explain the origin of the rhabdomyoblastic component: schwannoma cell metaplasia or mesodermal and ectodermal differentiation potential of neural crest cells. To the best of our knowledge, primary MTT of epididymis had not been reported in English literature before.

The clinical presentation of epididymis MTT is almost similar to benign tumor or malignant testicular tumor. Clinical signs were like any intrascrotal tumor. Accidental swelling mass may or may not be painful and occasionally accompanied by a hydrocele. These findings cannot distinguish a benign from a malignant tumor. The onset and duration of symptoms may provide helpful clues. The biological behavior of MTT was bad evolution with fatal ending. These neoplasms are generally asymptomatic but may have potentially life-threatening sequelae.

Diagnosis of MTT is difficult before surgery. In general, high-resolution scrotal ultrasonography remains the primary imaging method and may be extremely helpful in differential diagnosis of cystic or solid mass. Malignant tumors are either homogeneously hypoechoic or have a heterogeneous pattern of hypo- and hyperechoic areas [5]. However, US findings are often variable and nonspecific and do not usually allow definitive characterization. Computed tomographic and magnetic resonance imaging findings may define its relationship to various paratesticular structures in greater detail, which is necessary for clinical stages and help narrow the differential diagnosis [6]. When suspicious of malignancy, intraoperative frozen section analysis might be of great help.

The biphasic appearance of a pleomorphic spindle-cell tumor with scattered rhabdomyoblasts is highly suggestive of MTT; histological examination with immunohistochemical study was necessary for the diagnosis and differential diagnosis of this cancer. The neoplastic Schwann cells showed strong uniform immunoreactivity for S-100 protein. Neurofilament protein immunoreactivity was limited to the nerve fascicle on the tumor surface. Myoid markers such as desmin and MYO D1 showed no reactivity within the Schwann cells. In our case nerve sheath differentiation was confirmed by S-100 positivity and rhabdomyoblastic by positivity to desmin and MYOD1.

Given its rarity, the optimum local and systemic treatment for these tumors remains controversial. Most were treated according to standard protocol for managing soft tissue sarcomas, complete surgical resection is currently the mainstay of therapy, and radiotherapy should be considered routine regardless of reported margin status. Simple excision is inadequate, since wide repeat excision revealed microscopic residual disease in 27 % of completely excised cases [7]. Initial radical surgery including high ligation of the spermatic cord followed by adjuvant therapy is the current treatment of choice; high-dose radiotherapy is thought to be an alternative treatment could improve the therapeutical outcome [8]. Local recurrence should be treated by radiotherapy and metastases should be treated by chemotherapy [9]. Chemotherapy was reserved for palliation in the presence of advanced or metastatic disease. Standard chemotherapy does not appear to be of great benefit and novel therapies are required to improve outcomes.

There is a strong relationship of MTT with Neurofibromatosis type 1 and there is some controversy about how this affects outcome [10]. MTT is at a high risk of disease progression, the high risk of local recurrence demands long-term follow-up, elevated lactic dehydrogenase might also be a marker of retroperitoneal disease and poor prognosis. Improvement in survival requires effective systemic adjuvant therapy [11]. A multicenter study showed tumor site, size and grade represented the most important prognostic factors in genitourinary sarcomas [12].

## Conclusions

Malignant triton tumor has metastatic potential and poor outcome; histological criteria did not appear to accurately predict their clinical behavior and multimodality therapies are required. It should therefore be followed up for a long period after treatment.

## Consent

Written informed consent was obtained from the patient for publication of this case report and any accompanying images. A copy of the written consent is available for review by the Editor-in-Chief of this journal.
